# Transcriptomic comparison of the self-pollinated and cross-pollinated flowers of *Erigeron breviscapus* to analyze candidate self-incompatibility-associated genes

**DOI:** 10.1186/s12870-015-0627-x

**Published:** 2015-10-13

**Authors:** Wei Zhang, Xiang Wei, Heng-Lin Meng, Chun-Hua Ma, Ni-Hao Jiang, Guang-Hui Zhang, Sheng-Chao Yang

**Affiliations:** Yunnan Research Center on Good Agricultural Practice for Dominant Chinese Medicinal Materials, Yunnan Agricultural University, Kunming, 650201 Yunnan People’s Republic of China; The Life Science and Technology College, Honghe University, Mengzi, 661100 Yunnan People’s Republic of China; Key Laboratory of Tropical Agro-environment, Ministry of Agriculture, South China Agricultural University, Guangzhou, 510642 People’s Republic of China

**Keywords:** *Erigeron breviscapus*, Transcriptome, Self-incompatibility, Digital gene expression

## Abstract

**Background:**

Self-incompatibility (SI) is a widespread and important mating system that promotes outcrossing in plants. *Erigeron breviscapus*, a medicinal herb used widely in traditional Chinese medicine, is a self-incompatible species of Asteraceae. However, the genetic characteristics of SI responses in *E. breviscapus* remain largely unknown. To understand the possible mechanisms of *E. breviscapus* in response to SI, we performed a comparative transcriptomic analysis with capitulum of *E. breviscapus* after self- and cross-pollination, which may provide valuable information for analyzing the candidate SI-associated genes of *E. breviscapus.*

**Methods:**

Using a high-throughput next-generation sequencing (Illumina) approach, the transcriptionexpression profiling of the different genes of *E. breviscapus* were obtained, some results were verified by quantitative real time PCR (qRT-PCR).

**Results:**

After assembly, 63,485 gene models were obtained (average gene size 882 bp; N50 = 1485 bp), among which 38,540 unigenes (60.70 % of total gene models) were annotated by comparisons with four public databases (Nr, Swiss-Prot, KEGG and COG): 38,338 unigenes (60.38 % of total gene models) showed high homology with sequences in the Nr database. Differentially expressed genes were identified among the three cDNA libraries (non-, self- and cross-pollinated capitulum of *E. breviscapus*), and approximately 230 genes might be associated with SI responses. Several these genes were upregulated in self-pollinated capitulum but downregulated in cross-pollinated capitulum, such as *SRLK* (SRK-like) and its downstream signal factor, *MLPK*. qRT-PCR confirmed that the expression patterns of *EbSRLK1* and *EbSRLK3* genes were not closely related to SI of *E. breviscapus*.

**Conclusions:**

This work represents the first large-scale analysis of gene expression in the self-pollinated and cross-pollinated flowers of *E. breviscapus*. A larger number of notable genes potentially involved in SI responses showed differential expression, including genes playing crucial roles in cell-cell communication, signal transduction and the pollination process*.* We thus hypothesized that those genes showing differential expression and encoding critical regulators of SI responses, such as *MLPK*, *ARC1*, *CaM*, *Exo70A1, MAP, SF21* and *Nod,* might affect SI responses in *E. breviscapus.* Taken together, our study provides a pool of SI-related genes in *E. breviscapus* and offers a valuable resource for elucidating the mechanisms of SI in Asteraceae*.*

**Electronic supplementary material:**

The online version of this article (doi:10.1186/s12870-015-0627-x) contains supplementary material, which is available to authorized users.

## Background

Self-incompatibility (SI) is the most widespread and important mating system that promotes outcrossing while prevents inbreeding. Many flowering plants have SI system [1], which can be classified into two types: gametophytic SI (GSI) and sporophytic SI (SSI). The incompatibility phenotype of pollen is determined by its own haploid genotype in GSI, whereas it is determined by the diploid genotype of the parent plants in SSI [2]. The Brassicaceae is considered as a hotspot of SSI, which uses the pistil-expressed receptor kinase to recognize self/non-self-pollen [3] and is controlled by a multi-allelic *S* locus [4, 5]. *S* alleles have high amino acid sequence divergence within species [6–9]. *S*-locus receptor kinase (*SRK*) was identified as the female determinant [10]. *S*-locus cysteine-rich (*SCR*) proteins were identified as the male determinants in the Brassicaceae [11, 12]. Binding of SCR to SRK induces autophosphorylation of SRK, which triggers a signaling cascade leading to the SI response. Arm repeat containing 1 (*ARC1*) and *M*-locus protein kinase (*MLPK*) are two signaling molecules that positively mediate signal transduction. *ARC1* is expressed in the stigma and interacts with *SRK* through its cytoplasmic domain [13, 14]. *MLPK* was identified in a recessive mutant of *Brassica rapa* var. Yellow Sarson. Mutation of the gene leads to SI in *B. rapa* Yellow Sarson [15]. Other components associated with the SI signaling pathway include *thioredoxin-h 1* (*THL1*) and *thioredoxin-h 2* (*THL2*). Moreover, Ca^2+^ is also involved in signal transduction of SI. In *Citrus clementine*, several novel genes that potentially regulate Ca^2+^ homeostasis were identified during self-pollen recognition [16].

Genetic studies of SI in the Asteraceae started with species such as *Crepis foetida*, *Parthenium argentium* and *Cosmos bipinnatus* in the 1950s. Over 60 % of species in the Asteraceae are estimated to use SSI for genetic determination. First studies in these plants identified the SSI system in the Asteraceae and showed that the SI is controlled by the *S* locus [17, 18]. However, the precise number of *SRK* genes required for pollen specificity and the male and female determinants underlying SSI in the Asteraceae remains unknown. *Senecio squalidus* (Oxford Ragwort) has been used as a model plant to study the molecular mechanism of SI in the Asteraceae. Early studies on stigma surfaces in the Asteraceae indicated that the Asteraceae species have dry type stigmas. Later, Elleman et al. performed a comparative study of pollen-stigma interactions among five different plants and showed that stigmas of Asteraceae species produce a small amount of surface secretion and are not entirely dry [19]. This finding was subsequently confirmed by Hiscock et al. in *S. squalidus* and other Asteraceae species, which led to a reclassification of the Asteraceae stigma as ‘semi-dry’ [20]. It has been concluded that SSI in *Senecio* species operates through a different molecular mechanism with that in Brassica [21]. However, the *S. squalidus* dataset shared a greater number of homologous genes with the dry stigma species than the wet stigma species [22]. The number of *S* alleles in *S. squalidus* is low compared with other species that use SSI [23–25]. This inference was further analyzed through different transcripts of *S. squalidus* and SSH was successfully used to isolate pistil-enriched transcripts from three different S-genotypes of *S. squalidus*. 115 different candidates for pistil-specific genes in *S. squalidus* were identified [26]. Several new genes, such as *membrane associated protein* (*MAP*), *sunflower-21* (*SF21*) and *Nodulin/mtn3 gene* (*Nod*), might be linked with SI in *S.squalidus.* The *Senecio* pistil-specific *MAP* was found to be expressed in the papillar cells and transmitting tissue of the stigma [22, 27]. In *S. squalidus,* the nucleotide sequence of *MAP* exhibits relatively high *S*-genotypic polymorphism, which is elevated in the extracellular region [22, 27]. The *SF21* gene family was also isolated from *S. squalidus*. There are differences in these gene copies and expressed patterns [26]. *Nod* is expressed exclusively in the papillar cells of the *S. squalidus* stigma, where it appears to be developmentally regulated, reaching maximal expression as the stigmatic lobes reflex to expose the papillar cells [22, 27].

*Erigeron breviscapus* (Vant.) Hand.-Mazz, an important Chinese traditional medicinal plant [28, 29], is a self-incompatible species of Asteraceae. It has been used to treat cerebrovascular and cardiovascular problems [30]. Recently, accumulating studies on *E. breviscapus* have been focused on chemical components [31, 32], pharmacological activities [32–34], and germplasm resources [35–39]. However, little is known about the genetic mechanisms of SI responses in *E. breviscapus*.RNA sequencing is a powerful tool to investigate the molecular biology of many angiosperm and it has been used successfully for SI in lemon and *Leymus chinensis* [40, 41]. To uncover critical genes associated with SI responses, we compared the gene expression profiles in capitulum of *E. breviscapus* during non-, self- and cross-pollination, using transcriptome sequencing and *de novo* assembly. After analysis, a larger number of potential SI candidate genes showing differential expression were uncovered, including genes functioning in cell-cell communication, signal transduction and the pollination process*,* such as *MLPK*, *ARC1*, *CaM*, *Exo70A1, MAP, SF21* and *Nod.* Our study thus provides a pool of SI-related genes in *E. breviscapus* and offers a valuable resource to investigate the molecular mechanisms of SI responses in Asteraceae*.*

## Results

### Sequencing and de novo assembly of *E. breviscapus* transcriptome

**Fig. 1:**
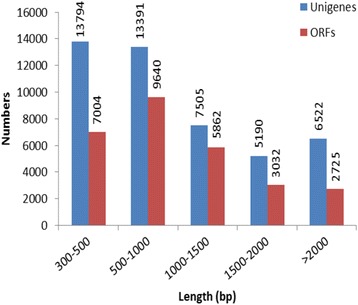
Length distribution of assembled unigenes and predicted ORFs

Three pools (self-pollinated, cross-pollinated and non-pollinated) capitulum of mRNA samples were used to build libraries for high-throughput sequencing using Illumina sequencing technology. The Illumina HiSeq 2000 next-generation sequencing generated 68.68 M raw reads comprising 13.87 Gb from the three libraries, with a Q30 percentage (sequencing error rate 0.1 %) above 82 %. The reads that only had 3′-adaptor fragments were removed from the raw reads, resulting in 53.44 M clean reads comprising 10.79 Gb with a Q30 above 94 % (Table 1). The filtered reads were de novo assembled by Trinity (kmer length = 25). The assembly results revealed that the transcriptome of *E. breviscapus* consists of 63,485 unigenes. The mean length of these unigenes was 882 bp and the N50 value was 1485 bp. The size distribution of the unigenes was shown in Fig. 1. The coding regions of each sequence were predicted by GetORF, which predicted 63,254 open reading frames (ORFs) from our assembled transcriptome, with 28,272 (44.7 %) ORFs longer than 300 bp. The mean length of these ORFs was 554 bp and the N50 value was 1167 bp. The size distribution of the unigenes is shown in Fig. 1. The high-quality reads produced in this study have been deposited in the NCBI SRA database (accession number: SRA245957).

**Table 1:** Quality of sequencing

Samples	Raw reads	Clean reads	Clean bases	Q30 %	GC %
T1 (control)	24,614,951	19,884,291	4,016,227,333	94.92	42.72
T2 (self-)	20,422,898	15,554,953	3,141,594,846	94.56	43.39
T3 (cross-)	23,642,295	18,001,474	3,635,693,053	94.69	43.30

### Functional annotation and classification

Annotation of the assembled unigenes was based on searches of specific databases for sequence similarity. All of the unigenes were compared with sequences in the Nr database, the Swiss-Prot protein database, the KEGG database and the COG database, using BLASTX with a cutoff e-value of 10^−5^. A total of 38,540 unigenes (60.70 % of all unigenes) returned a significant BLAST result (Additional file 1: Table S1). Among them, 38,338 unigenes (60.38 % of total unigenes) showed high similarity to sequences in the Nr database. The numbers of unigenes with significant similarity to sequences in the COG, KEGG and Swiss-Prot databases were 12,279 (19.34 %), 8483 (13.36 %), and 25,994 (40.94 %), respectively (Table 2).

**Table 2:** Summary of the annotation of unigenes of *E. breviscapus*

	Sequences	Frequency	≥300 nt	≥1000 nt
All assembled unigenes	63,485	100.00 %	46,402	19,217
Gene annotations against NR	38,338	60.38 %	33,418	18,211
Gene annotations against Swiss-Prot	25,994	40.94 %	23,024	13,451
GO annotation for NR protein hits	28,571	45.00 %	25,133	14,570
Gene annotations against COG	12,279	19.34 %	11,479	7,906
Gene annotations against KEGG	8,483	13.36 %	7,575	4,639
All annotated unigenes	38,540	60.70 %	33,547	18,231

**Fig. 2:**
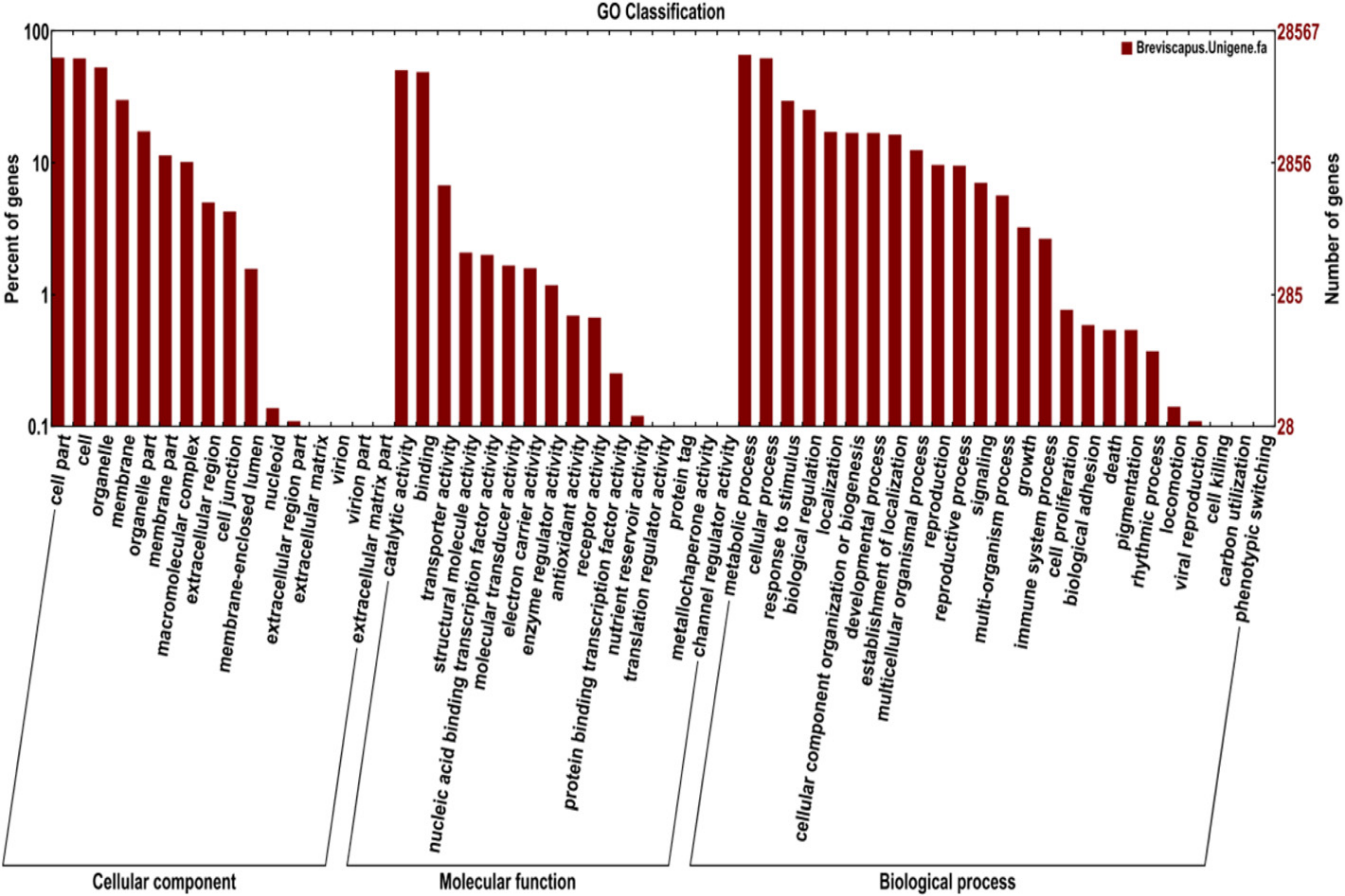
Gene Ontology classification of assembled unigenes. The 28,567 matched unigenes were classified into three functional categories: molecular function, biological process and cellular component

GO classification of the 28,571 annotated unigenes classified them into the functional categories: cellular components, molecular functions and biological processes. Among the various cellular components (ignoring unknown and other cellular component categories), cell, cell part and organelle were the most highly represented. Genes involved in other important cellular components, such as organelle parts, membrane, macromolecular complexes, extracellular regions, cell junction and membrane-enclosed lumen, were also identified through GO annotations. Similarly, binding and catalytic activities were most represented among various molecular functions; metabolic process and cellular process were most represented in the biological process categories (Fig. 2).

**Fig. 3:**
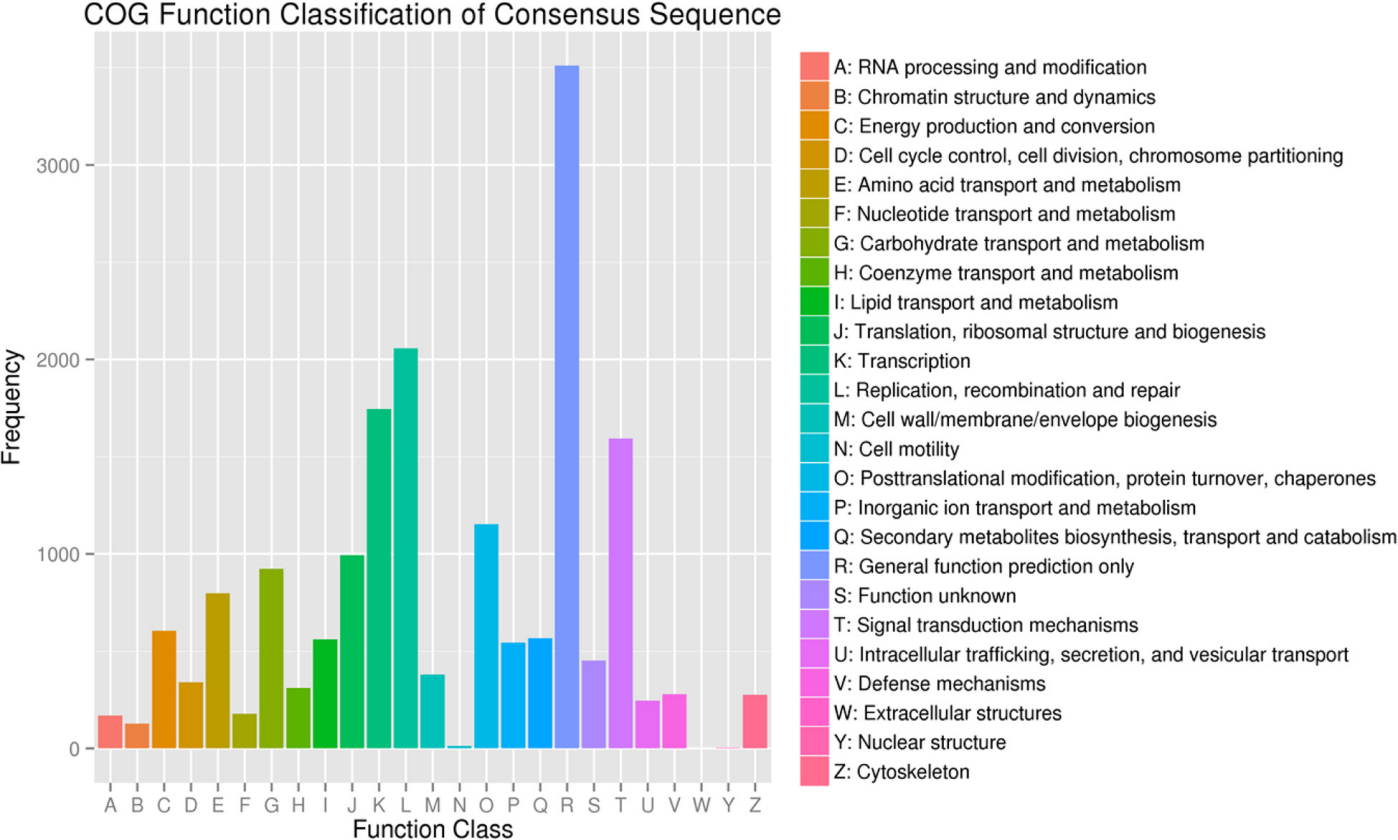
COG functional classification of all unigenes sequences. 12,279 (19.34 %) unigenes showed significant similarity to sequences in the COG databases and were clustered into 24 categories

COG is a database built on phylogenetic relationships of protein sequences from 66 genomes, including bacteria, plants and animals. Individual proteins or paralogs from at least three lineages are categorized in each COG to represent an ancient conserved domain. Within the *E. breviscapus* unigenes dataset, 12,279 were categorized (E-value ≤ 1.0E-5) into 25 functional COG clusters (Fig. 3). The five largest categories were: 1) General function prediction only; 2) Replication, recombination and repair; 3) Transcription; 4) Signal transduction mechanisms; and 5) Post-translational modification, protein turnover and chaperones.

### Gene expression differences in the three libraries

Gene expression level was calculated using the RPKM method, which takes the influence of both the sequencing depth and gene length on read count into account. On the basis of the applied criteria [FDR ≤ 0.01 and log_2_ (fold-change) ≥ 1], 2072 genes were identified as significantly differentially expression genes (DEGs) between sample T1 (non-pollinated flowers) and T2 (self-pollinated flowers), in which 1426 were upregulated genes and 646 were downregulated in the *E. breviscapus* transcriptome. In addition, 2099 genes were identified as significant DEGs between samples T1 and T3 (cross-pollinated flowers), and 145 genes were identified as significant DEGs between samples T2 and T3. For these DEGs, GO and KEGG analyses were performed.

**Fig. 4:**
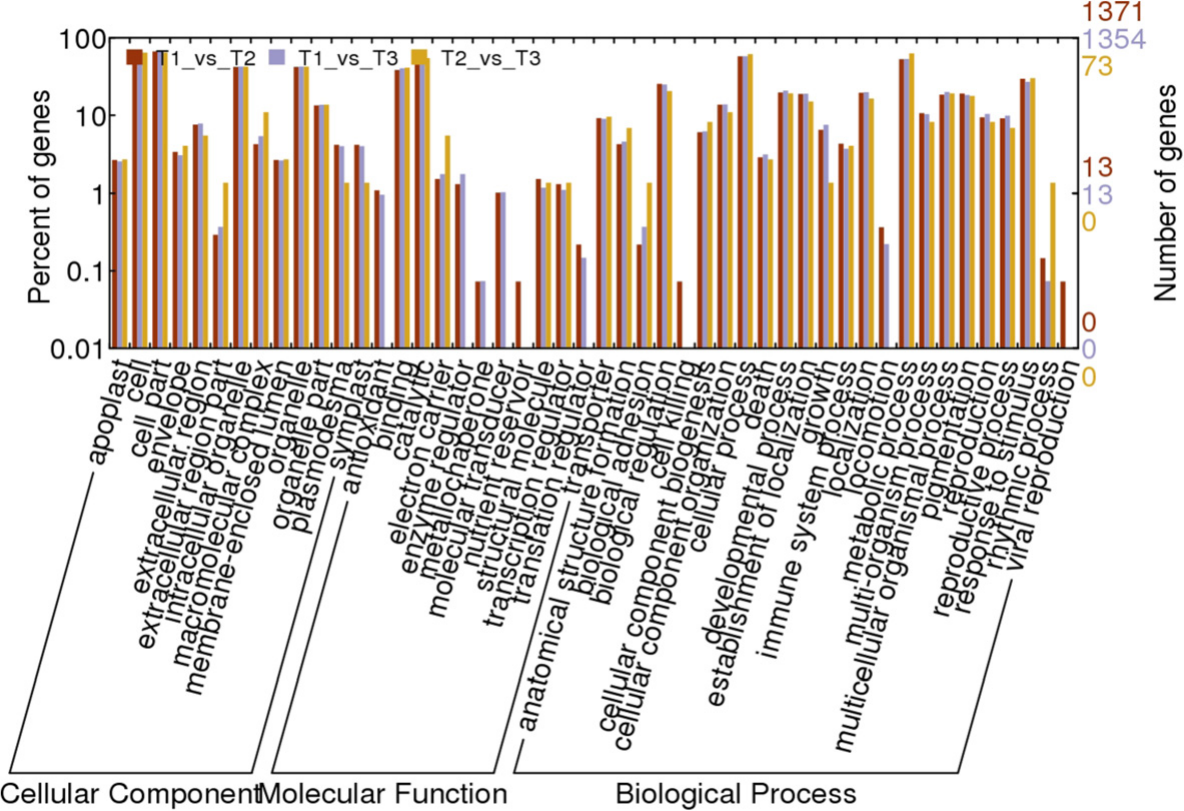
Gene Ontology classification of differentially expressed genes between samples

**Fig. 5:**
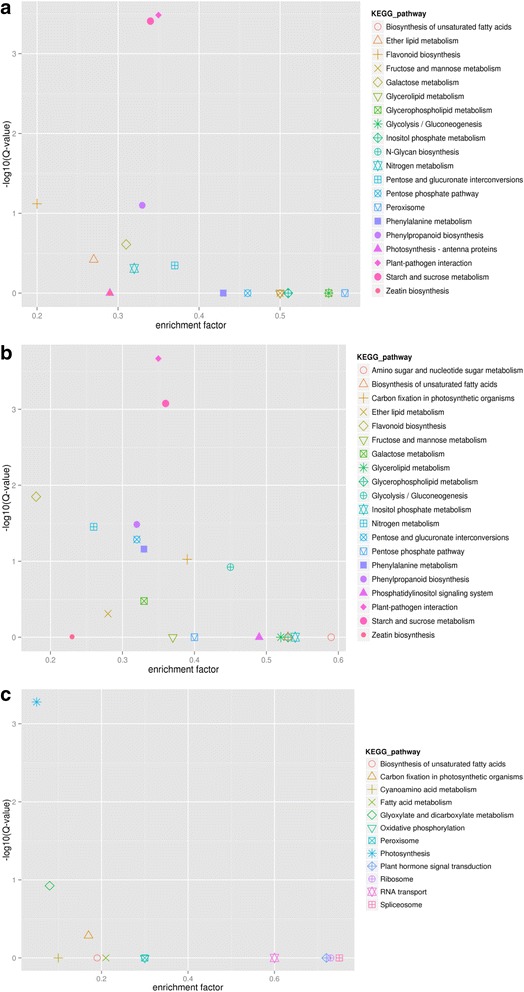
**a**, **b** and **c** Scatterplot of differentially expressed genes enriched in KEGG pathways. The rich factor represents the ratio of the number of DEGs and the number of all the unigenes in the pathway; the Q value represents the corrected *P*-value

Between samples T1 and T2, 1371 DEGs were associated with 50 subcategories, which were grouped into three major categories: biological processes, cellular components and molecular functions. In each of the three major categories of the GO classification, ‘metabolic process’, ‘cell part’ and ‘catalytic activity’ terms were dominant (Fig. 4). To further investigate the biochemical pathways of these DEGs, we mapped all DEGs to terms in the KEGG database. Of the 2072 DEGs, 220 genes had a KO ID and could be categorized into 78 pathways. Of those, two pathways were significantly enriched (corrected P value ≤0.05): genes involved in plant-pathogen interaction and starch and source metabolism being the most significantly enriched (Fig. 5a). Between samples T1 and T3, the expression levels of 1452 genes were upregulated and 647 genes were downregulated; 1354 of these DEGs were associated with 52 sub-categories, and 230 were mapped to 81 pathways (Fig. 5b). Between samples T2 and T3, the expression levels of 92 genes were upregulated and 53 genes were downregulated; 73 of these DEGs were associated with 35 sub-categories, and 14 were mapped to 12 pathways (Fig. 5c).

### Comparison of transcriptional profiles of genes associated with SI responses among the three libraries

**Fig. 6:**
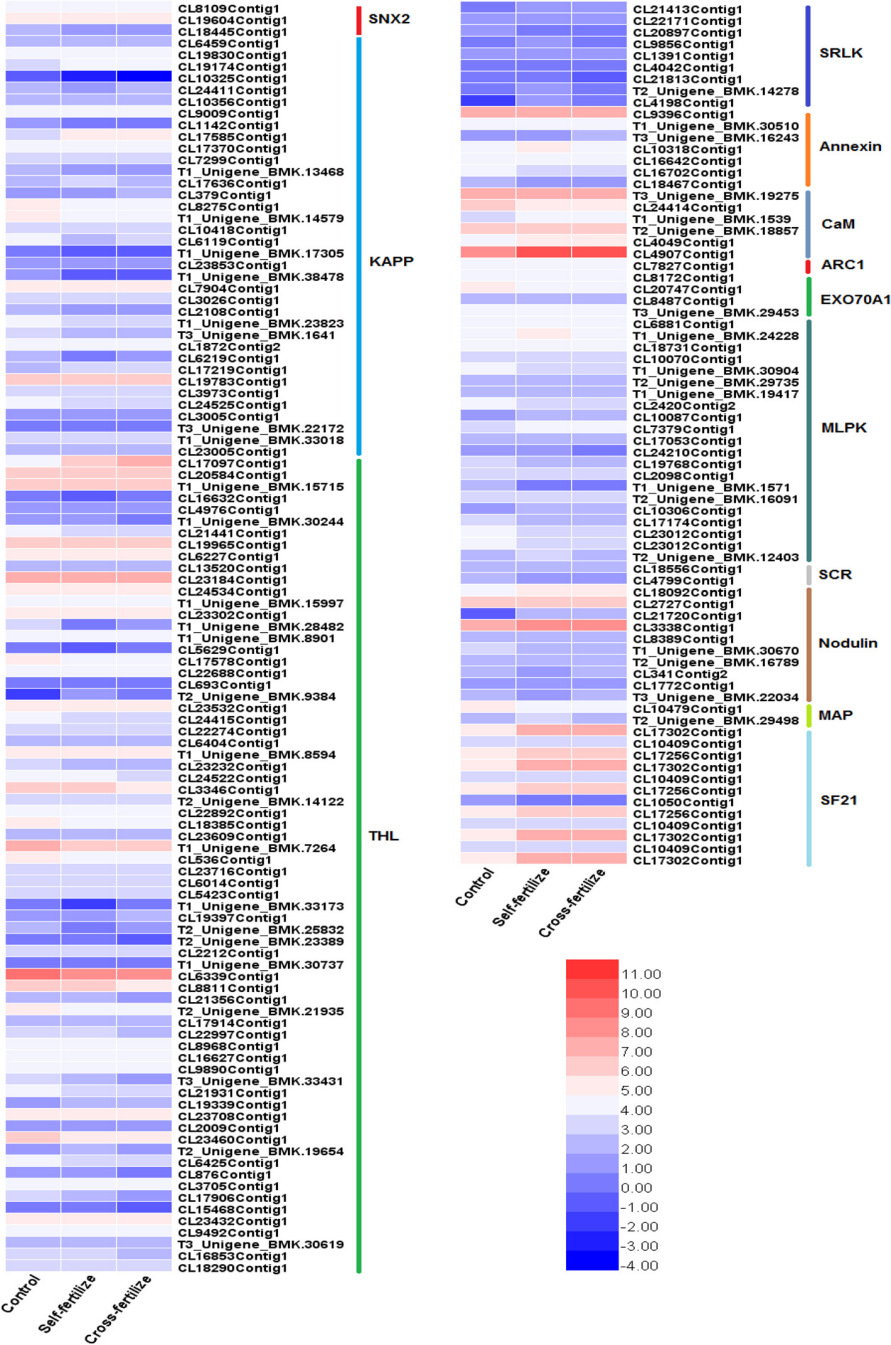
**a** and **b** Heatmap analysis of three *E. breviscapus* samples for differentially expressed genes involved in SI, based on gene ontology analysis. *Red shades* indicate higher expression and *green shades* indicate lower expression. The *color key* indicates the intensity associated with normalized expression values

Previous researches demonstrated that a number of genes involved in SI responses are differentially expressed, such as genes for cell-cell communication and signal transduction. To uncover genes involved in SI responses in *E. breviscapus*, the relative expression levels of SI-associated genes were analyzed in detail and the results demonstrated that most of their unigenes showed significant changes in expression levels in the three libraries. Those genes associated with SI were clustered according to similarities in expression profiles between self- and cross-pollination. A heat map of gene expression of 230 putative genes involved in SI by pheatmap software is shown in Fig. 6. Variance-stabilized data obtained using the DESeq package was used to generate the heat map by pheatmap software. The RNA-seq analysis of transcriptional data revealed that some genes (*SRLK, MLPK* and *KAPP*) were highly expressed in self-pollinated capitulum but poorly expressed in cross-pollinated ones. In contrast, *THL* and *CaM* were downregulated after self-pollination but upregulated after cross-pollination. However, *Exo70A1* showed no difference among three cDNA libraries. These genes will be further examined to investigate their biological functions. The RPKM value of partial unigenes involved in SI responses were listed in Additional file 2: Table S2.

### Identification and expression analysis of candidate genes involved in SI responses

**Fig. 7:**
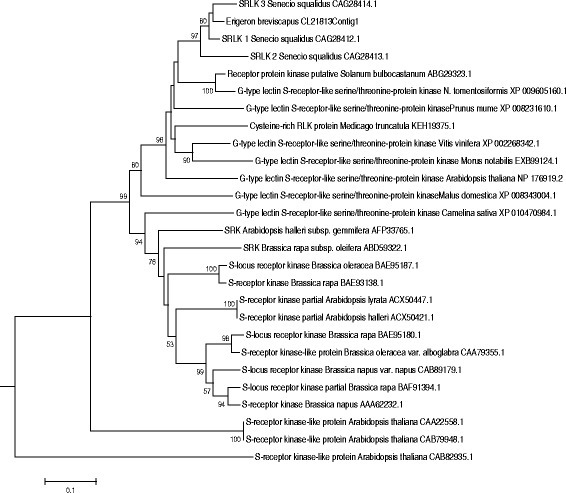
Phylogenetic analysis of CL21813contig1 from *E. breviscapus* with other *SRKs* from different species. The neighbor-joining tree based on p-distance and pairwise deletions of gaps indicates the phylogenetic relationships among the putative SRK proteins in different species. The numbers at the branching points indicate the bootstrap support values (200 replicates). The putative *E. breviscapus S* locus receptor protein kinase is marked with a triangular symbol

**Fig. 8:**
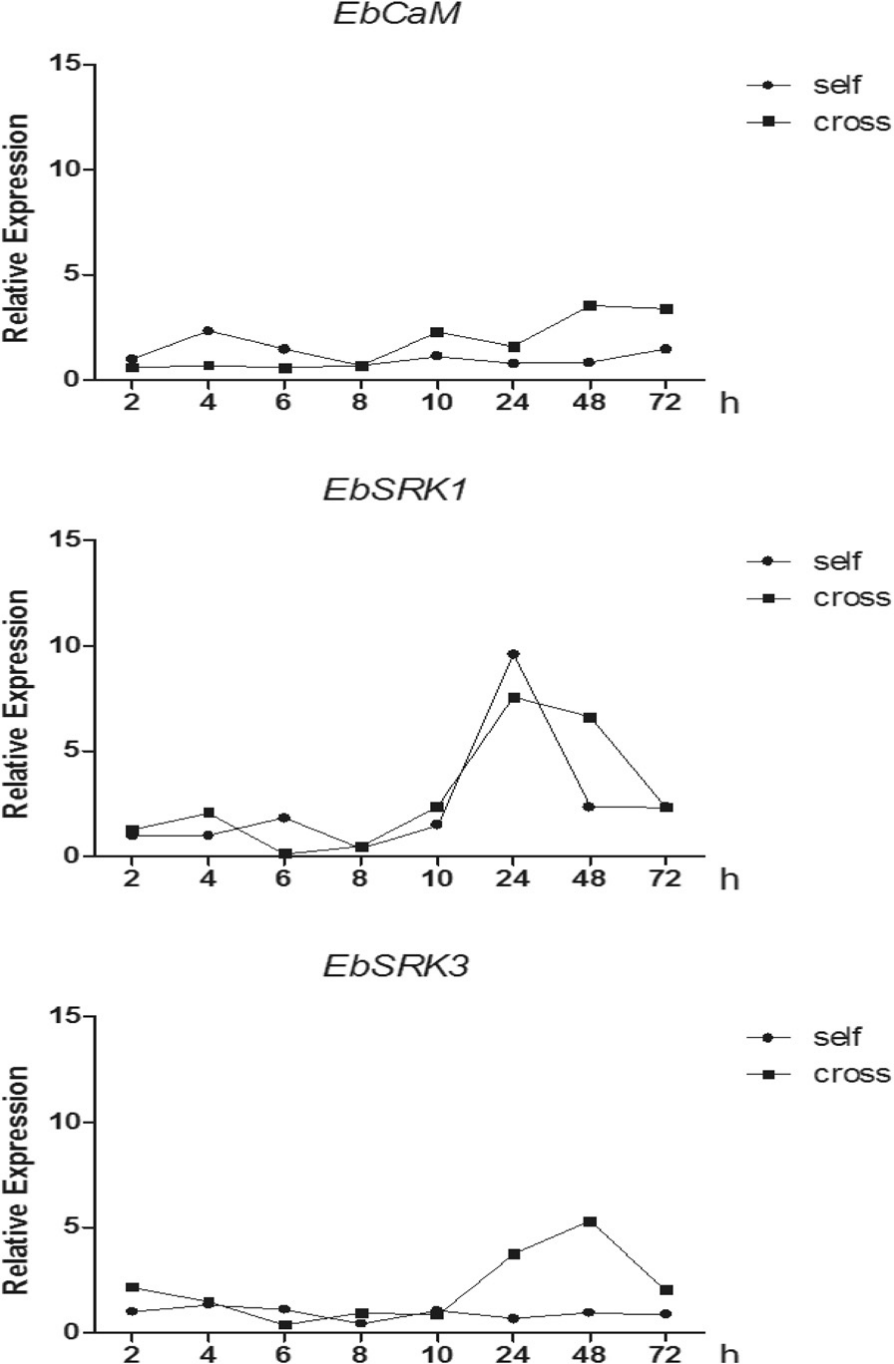
Relative gene expression of selected genes during the pollen-pistil interaction. Relative expression was defined as the expression level and the *x*-axis indicates hours after pollination. CL21813contig1 (*S*-locus receptor kinase1, *EbSRLK1*); T3_Unigene_BMK.9975 (*S*-locus receptor kinase3, *EbSRLK3*); CL4907Contig1 (calmodulin). Styles with capitulum were sampled from 2 to 72 h

A putative *SRK-like* gene, *EbSRLK 1* (CL21813Contig1), was cloned. Phylogenetic analysis was performed by alignment using entire predicted protein sequences from *E. breviscapus* and other species by mega software. The neighbor-joining phylogenetic tree demonstrated that CL21813contig1 (*EbSRLK1*) is most closely related to *SRK* from *S. squalidus* L., but is distant from the *SRK* of crucifer species (GenBank accession numbers: CAG28412, CAG28414 and CAG28413) (Fig. 7). Calcium ions are able to transmit diverse signals that exert primary actions on cells. *Calmodulin* (*CaM*), as the multifunctional calcium receptor, is associated with various physiological and developmental processes in plants. The RNAseq results showed that the expression levels of CL21813contig1 (*EbSRLK1*) and T3_Unigene_BMK.9975 (*EbSRLK3*) were upregulated after self-pollination. The highest expression levels were observed at 24 and 10 h after self-pollination for *EbSRLK1* and *EbSRLK3*, respectively. However, their expression levels were lower in cross-pollinated capitulum than in self-pollinated capitulum. The highest expression levels for *EbSRLK1* and *EbSRLK3* in cross-pollinated capitulum were observed at 24 and 48 h after pollination, respectively. CL4907Contig1 (*EbCaM*) was downregulated after self-pollination and upregulated after cross-pollination. The expression levels of the three genes were significantly different between self- and cross-pollination (repeated-measures ANOVA: *EbSRLK1* time effect, *F*_1,16_ = 96.822, *P* < 0.001. time * treatment, *F*_7,16_ = 100.492, *P* < 0.001; treatment, *F*_7,16_ = 34.321, *P* < 0.001; *EbSRLK3* time effect, *F*_1,16_ = 16.929, *P* = 0.001. time * treatment, *F*_7,16_ = 212.676, *P* < 0.001; treatment, *F*_7,16_ = 112.076, *P* < 0.001; *EbCaM* time effect, *F*_1,16_ = 14.695, *P* = 0.001. time * treatment, *F*_7,16_ = 18.906, *P* < 0.001; treatment, *F*_7,16_ = 20.065, *P* < 0.001). Furthermore, the expressions of the three genes were significantly different at 6, 10, 24 and 48 h after self- and cross-pollination (*EbSRLK1* independent *T*-test: *t* ≥ 5.650, *P* ≤0.005; *EbSRLK3 t* ≥9.740, *P* ≤0.001; *EbCaM t* ≥6.208, *P* ≤0.003). To confirm the results of RNAseq, three candidate SI-associated genes (two *EbSRLKs* and one *CaM*) were chosen for further expression analysis. Using gene-specific primer pairs, the expression levels of the candidate genes were analyzed at different time points after self- or cross-pollination by using qRT-PCR (Fig. 8). The expression patterns of these three genes in the qRT-PCR analysis showed the similar trend as the RNAseq analysis (Fig. 8).

## Discussion

To identify genes involved in the SI, we sequenced the *E. breviscapus* transcriptome and de novo assembled reads from next-generation RNA-seq data. We identified 63,485 unigenes in the three libraries (Sample T1 for non-pollinated flowers, Sample T2 for self-pollinated flowers, and Sample T3 for cross-pollinated flowers). In the absence of *E. breviscapus* genomic information, the availability of the transcriptome data will provide a valuable resource to investigate the mechanisms of SI responses.

### Genes involved in SI responses in *E. breviscapus*

The *S*-locus encodes two proteins: SRK and SCR. *SRK* encodes an *S* receptor kinase [42, 43]. *E. breviscapus* exhibits an SSI system of SI as other Asteraceae species. *S* alleles have high amino acid sequence divergence within species, and *E. breviscapus* is no exception: the pistil *SRLK* genes from the transcriptome showed sequence divergence. Phylogenetic analysis of these unigenes with other *SRKs* from different species demonstrated that the characteristics of some *SRKs* (such as T1_unigene_BMK.9975 and CL21413Contig1) in *E. breviscapus* are not consistent with other *SRK* genes (not shown). The RNA-seq analysis of transcriptional data revealed the same expression pattern (Fig. 6b). Previous studies of *S. squalidus* have shown that orthologues of the *Brassica S*-gene, *SRK*, are not expressed exclusively in the stigma, or linked to the *S*-locus [21]. In the three samples, differentially expressed, putative *EbSRLK* genes were identified. One putative *EbSRLK1* gene (CL21813contig1) was cloned. Quantitative PCR analysis revealed that *EbSRLK1* was highly expressed in self-pollinated capitulum and poorly expressed in cross-pollinated ones (Fig. 8). However, their expression patterns suggested that they are unlikely to be directly involved in SI and which may be similar to the *SRK-like* genes in *S. squalidus*. Further studies are needed to dissect the functions of these *SRK-like* genes in *E. breviscapus*.

In contrast to the female factor *SRKs*, the male *S*-determinant SCR acts as a ligand [44]. The HV region in the *S*-domain of SRKs acts as the SCR-binding site, which involves a two-stage recognition process [45]. In the three libraries of *E. breviscapus*, only two unigenes were annotated as SCR, CL18556Contig1 and CL4799Contig1 (Fig. 6b). Functional analysis of the role of SCRs in *E. breviscapus* during pollen-pistil interaction will be performed in a future study.

### Signaling pathway for the SI reaction

The *E. breviscapus* dataset revealed many genes that play primary roles in the recognition between the stigma and pollen grains. Among the identified genes, we focused on those genes encoding *MAP*, *SF21*, *Nodulin3*, *ARC1*, *MLPK*, *Exo70A1*, *CaM* and *THL1*/*THL2*.

*ARC1* and *MLPK* are candidate downstream effectors of *SRK*. therefore, *ARC1* might be a positive effector in the SI response in *Brassica*. Antisense suppression of *ARC1* led to partial breakdown of the SI response [46]. In the three samples in this study, only one *ARC1* transcript was found (Fig. 6b). The absence of other *ACR1* genes suggest that the assemblies were incomplete and/or that expression of the *ACR1* genes is too low to acquire enough reads to be assembled. *MLPK* can interact with the kinase domain of *SRK* [47]. *MPLK* is thought to function in SRK-mediated signaling. In the present study, we found that the *MLPK* gene was upregulated in the self-pollinated and downregulated in cross-pollinated sample (Fig. 6b).

As a factor interacting with *ARC1*, *Exo70A1* has been isolated in *B. napus* [48]. Overexpression of *Exo70A1* in self-incompatible *B. napus* partially breaks down SI, whereas suppression of *Exo70A1* (by RNAi or T-DNA insertion) in self-compatible *B. napus* and *A. thaliana* inhibited pollen adhesion, hydration and germination [48]. Six unigenes encoding *Exo70A1* were identified in our study, although no differences in their expressions were observed among three cDNA libraries (Fig. 6b). Further studies are required to clarify the function of *Exo70A1* genes in *E. breviscapus*.

*THL1* and *THL2* were identified as SRK-binding partners from a yeast two-hybrid screen [49] and which act as negative regulators in SRK signaling [50, 51]. Studies on the Brassicaceae showed that THL plays a key role in SI response. In the three cDNA libraries of *E. breviscapus*, one striking finding was the identification of 88 putative THL proteins (Fig. 6a). Functional analysis will be necessary to shed light on the role of the *THL1*/*THL2* during signal transduction in *E. breviscapus*.

Calmodulin is an important second messenger in many signal transduction pathways and an important calcium-banding protein. CL4907Contig1 (*EbCaM*) was downregulated after self-pollination and upregulated after cross-pollination (Fig. 8). Quantitative PCR analysis showed that the expression pattern of the *EbCaM* gene was the same as in the RNA-seq analysis (Fig. 6a and b).

Some new SI candidate genes, *MAP*, *SF21*, *Nod*, were identified based on the fact that they specifically express in pistils of *S. squalidus*. The nucleotide sequence of *MAP* exhibits relatively high *S*-genotypic polymorphism [22]. In the three samples in *E. breviscapus*, few *MAP* transcript was found. The reason for the deletion of *MAP* gene may be similar to *ARC1* gene, i.e., the assemblies were incomplete and/or that expression of the *MAP* genes is too low to acquire enough reads to be assembled (Fig. 6b). Phylogenetic analysis of *SF21* nucleotide sequence alignments indicate that this gene family is conserved and may play an important role in reproductive processes in flowering plants. In *E. breviscapus* transcriptome dataset, we found that the *SF21* genes were upregulated in the self-pollinated and cross-pollinated sample (Fig. 6b). *Nod* gene family has been investigated in Arabidopsis and rice, which have a crucial function in pollen development [52, 53]. Despite the large number of pistil-specific *nodulin/mtn3* genes have been investigated, the function of these genes in pistils has not been studied. Ten unigenes encoding *Nod* were identified in our study, they were also upregulated in the self-pollinated and cross-pollinated sample (Fig. 6b). *SF21* and *Nod* might well be involved in SI, however, further studies are needed.

Moreover, there were many other genes that might be involved in SI in the three libraries of *E. breviscapus*, such as SNX2, KAPP and Annexin (Fig. 6a and b). Further studies are required to fully understand the role and mechanism of these candidate genes in SI.

## Conclusions

We performed the first large-scale investigation of gene expression in the capitulum of *E. breviscapus*, an Asteraceae SSI species, using high-throughput RNA-seq analysis. After assembly, 63,485 gene models were obtained (average gene size 882 bp; N50 = 1485 bp), among which 38,540 unigenes (60.70 % of total gene models) were annotated by comparing them with four public databases (Nr, Swiss-Prot, KEGG and COG). 38,338 unigenes (60.38 %) showed high similarity with sequences in the Nr database. DEGs were identified among the three cDNA libraries (non-, self- and cross-pollinated capitulum of *E. breviscapus*). Approximately 230 genes showed differential expression that might be associated with SI. Quantitative PCR confirmed the expression patterns of selected genes examined by RNA-seq. Most of the genes identified by the RNA-seq analysis were not previously reported or studied in *E. breviscapus*. Although function information is missing, we hypothesized that *MLPK*, *ARC1*, *CaM*, *Exo70A1, MAP, SF21* and *Nod* might be crucial for the SI responses in *E. breviscapus.* However, *EbSRLK1* and *EbSRLK3* genes were found not closely related to SI in *E. breviscapus* and they are more like the *SRK-like* genes. The results will lead to a better understanding of the SSI system in the Asteraceae. The functions of these genes will provide clues to the mechanisms that underlie SI in *E. breviscapus* SSI systems.

## Methods

### Plant materials and RNA isolation

Wild-type of *E. breviscapus* were used in this study. Flower tissues of *E. breviscapus* were collected from an experimental plot in the planting base (Luxi County, Yunnan, China). We have got permission from the sample provider. Experiments of forced self-pollination and cross-pollination were performed at 3rd April 2014. In short, plants were prepared for experiments by bagging branches bearing developing flower buds. All pollinations (selfs and crosses) were repeated for at least three times and at least ten bagged flowering heads (capitula) were collected. Controlled self- and cross-pollinations were performed as previously described in Hiscock [54] and Brennan et al. [23, 25]. In cross treatment, the anthers were removed by a sable paint brush. Cross-pollinations were carried out by touching stigmas with a capitulum containing flowers with pollen fully presented. Following pollination, capitula were secured within a small, porous, tissue pollination bag (5 cm x 5 cm) using a pin. On the other hand, in forced-selfing, selfing solution (1 % NaCl in 10.1 % Tween 20) was applied to stigmas of bagged capitula with a dry paint brush. Stigmas were allowed to dry for some times, then applying self-pollen from another capitulum from the same individual and re-bagging selfed capitula. Self-pollination was enhanced by occasional agitation of bagged capitula. Three samples were harvested at 24 h after non- (Sample T1), self- (Sample T2) and cross-pollination (Sample T3).

For Illumina sequencing, total RNA was extracted using an EASY spin microRNA Rapid extraction Kit (Aidlab, Beijing, China). Both the quantity and quality of the total RNA were verified using a NanoDrop ND1000 (Thermo Scientific), gel electrophoresis and an Agilent 2100 Bioanalyzer (Agilent). Thirty micrograms of RNA was pooled equally from 10 capitulum for cDNA library preparation.

### RNA-seq library construction and sequencing

The cDNA library was constructed using a cDNA Sample Preparation Kit (Cat # RS-930-1001, Illumina Inc., San Diego, CA), following the manufacturer’s instructions. The total RNA was collected and pooled from each treatment, and mRNA was enriched and purified using poly-T oligo nucleotide-attached magnetic beads. The mRNA was cleaved using an RNA Fragmentation Kit (Ambion, Austin, TX, USA) and then used as templates for first-strand cDNA synthesis using reverse transcriptase (Invitrogen, Carlsbad, CA, USA) and random hexamer primers. Second-strand cDNA was synthesized using DNA polymerase I (New England BioLabs, Ipswich, MA). The cleaved fragments were purified using a MinElute PCR Purification Kit (Qiagen, Dusseldorf, Germany) and ligated to Solexa adaptors. The desired fragments (ca. 150–200 bp in size) were excised from 1.8 % agarose gels and purified using a Gel Extraction Kit (Qiagen). Finally, PCR amplicons were purified and the sequencing library was successfully constructed. The Illumina HiSeq™2000 sequencing platform was used for sequencing. The transcriptome datasets were analyzed by Illumina software.

### De novo transcriptome assembly

After sequencing, raw reads that contained adapters, reads containing more than 5 % of unknown sequences (‘N’), and low-quality bases that were identified based on CycleQ30 (corresponding to a 0.1 % sequencing error rate) were removed before assembly. De novo assembly of the clean reads was performed using the Trinity program [55]. First, reads with a certain length of overlap were combined to form contigs. They were then mapped back to contigs with paired-end reads to detect contigs from the same transcript, as well as the distances between contigs. Finally, Trinity connected contigs and obtained sequences that could not be extended on either end. Such sequences were defined as ‘unigenes’. After that, unigenes from the three libraries were further assembled to obtain non-redundant unigenes using the TGICL software and non-redundant unigenes were used for further analysis [56]. Potential coding regions within the unigenes were analyzed using the GetORF function in the EMBOSS software package. Length distribution was analyzed using common perl scripts. The N50 length, mean length and unigene number with different length intervals were all calculated.

### Functional annotation and KEGG pathway analysis

The functions of the unigenes were annotated using BLASTX [57] searches with an E-value threshold of 10^−5^ against protein databases, including the NCBI non-redundant (Nr) database (http://www.ncbi.nlm.nih.gov) [58], the Swiss-Prot protein database (http://www.expasy.ch/sprot) [59], the Kyoto Encyclopedia of Genes and Genomes (KEGG) database (http://www.genome.jp/kegg) [60], and the Clusters of Orthologous Groups of proteins (COG) database (http://www.ncbi.nlm.nih.gov/COG) [61]. The proteins with the highest sequence similarity were retrieved for analysis. KEGG produced annotations of metabolic pathways, while COG matched each annotated sequences to a conserved domain.

### Abundance estimation and differential expression analysis

To investigate the expression level of each unigene in different samples, transcript abundance estimates were obtained by running RNA-seq by Expectation-Maximization analysis separately for each sample, which uses an iterative process to fractionally assign reads to each transcript based on the probabilities of the reads being derived from each transcript [62]. The alignment produced digital expression levels for each unigene, which were normalized using perl scripts in the Trinity package to obtain reads per kilo base per million (RPKM) values [63].

To study the expression patterns of the transcripts across samples, it is often useful to restrict analysis to those transcripts that are significantly differentially expressed in at least one pairwise sample comparison. Differential expression analysis of the three samples was performed using tools from the Bioconductor project, including edgeR [64] and DESeq [65], to identify differentially expressed transcripts. Given a set of differentially expressed transcripts, we extracted those transcripts with an FDR (false discovery rate) ≤0.01 and log_2_fold-change (log_2_FC) ≥1.

### Gene validation and expression analyses

To investigate different gene (*EbSRLK1*, *EbSRLK3* and *EbCaM*) expressions in *E. breviscapus*, eight flower stages (2, 4, 6, 8, 10, 24, 48 and 72 h after pollination) were collected and examined. qRT-PCR was performed with gene-specific primers. All primers used in this study are listed in Table 3. Total RNA was extracted from self-pollinated, cross-pollinated, or non-pollinated (control) capitulum in *E. breviscapus* using the TRIzol Reagent (Takara, Beijing, China). RNA was further purified using an RNA purification kit (Takara). In this experiment, GAPDH was chosen as the housekeeping gene. Each reaction was performed in triplicate (each biological replicate contained tissues from at least ten capitulum) and the experiment was repeated three times (technical replicates) for each sample. The standard curve method was used to determine the mRNA relative expression levels, which were normalized to the reference gene. Real-time PCR was performed in a Roche detection system (Roche, Switzerland). The thermal cycler parameters were used as follow: 30 s at 94 °C; then 45 cycles of 20 s at 94 °C, 20 s at 55 °C, and 30 s at 72 °C. The melting curve analysis was carried out from 60 °C to 95 °C to observe the specificity of the PCR products. Data were analyzed using repeated-measures ANOVA, using time (2, 4, 6, 8, 10, 24, 48 and 72 h) as the within-subject effect and different mating patterns (self- and cross-pollination) as the between-subject effect, respectively. Independent t-tests were performed to explore the detailed effects of treatment. All calculations were performed using SPSS Statistics 17.0 (www.spss-china.com).

**Table 3:** Primers used for gene validation and expression analyses

Primers	Sequences	Size (bp)
EbSRLK1F	GGGCAGAATCGGACCCTTAC	95
EbSRLK1R	TGAGACCCTTCTTCGTTGG	95
EbSRLK3F	CTAGGCTGTTGCATTCGTGG	293
EbSRLK3R	GGTGATTGCTTCAGTCTCATTTGG	293
EbCaM-F	AGGCTGAACTCCAAGACAT	118
EbCaM-R	TCCTCCTCAGAGTCCGTAT	118

## Availability of supporting data

The supporting data such as the high-quality reads produced in this study have been deposited in the NCBI SRA database, an open access repository (accession number: SRA245957).

## Additional files

Additional file 1:
**Table S1.** Functional annotation of *E.breviscapus* transcriptome. (XLSX 4281 kb) Additional file 2:
**Table S2.** The RPKM value of partial unigenes. (XLSX 520 kb)

## Abbreviations

SI: Self-incompatibility
GSI: Gametophytic self-incompatibility
SSI: sporophytic self-incompatibility
ORF: Open reading frames
RPKM: Reads Per Kilobase of exon model per Million mapped reads
FDR: False discovery rate
DEGs: Differentially expression genes
BLAST: Basic Local Aligment Search Tool
Go: Gene Ontology
KEGG: The KEGG resource for deciphering the genome
COG: Clusters of orthologous Groups of proteins
Nr: ncbi nr database
qRT-PCR: Quantitative real time polymerase chain reaction
*E. breviscapus*: *Erigeron breviscapus*
SRLK: *S*-locus receptor-like kinase
SCR: *S*-locus cysteine-rich
*MLPK*: *M*-locus protein kinase
*ARC1*: Arm repeat containing 1
*CaM*: Calmodulin
*MAP*: Membrane associated protein
*SF21*: *Sunflower-21*
*Nod*: *Nodulin/mtn3*
*THL1*: Thioredoxin-like protein 1
*THL2*: Thioredoxin-like protein 2
KAPP: Kinase associate protein phosphatase
SNX2: Annexin S2
Swiss-Prot: A high quality annotated and non-redundant protein sequence database
edger: a Bioconductor package for differential expression analysis of digital gene expression data Bioinformatics
DESeq: Different gene expression analysis based on the negative binomial distribution
FDR: False discovery rate
